# Study of the In Vitro Antagonistic Activity of Various Single-Strain and Multi-Strain Probiotics against *Escherichia coli*

**DOI:** 10.3390/ijerph15071539

**Published:** 2018-07-20

**Authors:** Sabina Fijan, Dunja Šulc, Andrej Steyer

**Affiliations:** 1 Faculty of Health Sciences, University of Maribor, Žitna ulica 15, 2000 Maribor, Slovenia; dunja.sulc@student.um.si; 2 Faculty of Medicine, Institute of Microbiology and Immunology, University of Ljubljana, Zaloška 4, 1000 Ljubljana, Slovenia; andrej.steyer@mf.uni-lj.si

**Keywords:** probiotic, *Escherichia coli*, antagonistic effect

## Abstract

*Escherichia coli* is an important commensal of our gut, however, many pathogenic strains exist, causing various severe infections in the gut or beyond. Due to several antibiotic resistance patterns of *E. coli*, research of alternative treatments or adjuvant therapy is important. One of these is the use of probiotics as antagonistic agents against *E. coli*. Most published studies investigate only one strain of *E. coli* and single-strain probiotics. The objectives of this study were to evaluate the antagonistic activity of selected single-strain and multi-strain probiotic supplements against selected clinical *E. coli* pathotypes using the in vitro agar spot test and the co-culturing method. Molecular methods were used to determine the presence of the genus lactobacilli and bifidobacteria as well as certain selected strains in the probiotic supplements. The agar-spot test showed that the multi-strain probiotics were more effective than the single-strain probiotics. On the other hand, the co-culturing method showed the opposite result, indicating that results are importantly influenced by the chosen method. The most effective single-strain probiotics against *E. coli* strains were *Bifidobacterium animalis* subsp. *lactis* BB-12 and *Lactobacillus reuteri* DSM 17938. The most effective multi-strain probiotics contained lactobacilli, bifidobacteria and enterococci strains, thus proving that most effective probiotics against *E. coli* strains are the lactic acid bacteria and bifidobacteria. The overall results from both in vitro tests reveal that all selected probiotics exhibited an antagonistic activity against all *E. coli* strains. From a public health perspective probiotics have thus proved to be successful in inhibiting the growth of *E. coli* and could therefore be used as adjuvant therapy or alternative therapy in *E. coli* infections.

## 1. Introduction

*Escherichia coli* is facultative anaerobe that is generally considered a commensal inhabitant or resident bacteria in lower numbers of the gastrointestinal tract of humans and warm-blooded animals as well as cold-blooded animals [1,2]. It is also one of the bacteria that colonizes the infant gut and aids in establishing an anaerobic environment which enables a shift to the colonisation of obligate anaerobes, of which bifidobacteria are the largest group in the infant microbiome [3,4]. In order to become a resident of the human gastrointestinal tract *E. coli* must evade host defenses, survive acidity in the stomach, acquire nutrients for survival, compete with other microorganisms and adhere to the gut epithelium by entering the mucus layer. Certain strains of *E. coli*, however, are some of the important causes of healthcare-associated infections and carry several virulent factors and can easily gain resistance to antibiotics, which include resistance caused by extended-spectrum *β*-lactamases (ESBLs) [5,6]. The so-called diarrheagenic *Escherichia coli* (DEC) or intestinal group includes the following subgroups: enterotoxigenic *E. coli* (ETEC), Shiga toxin-producing *E. coli* (STEC), enteroinvasive *E. coli* (EIEC), enteropathogenic *E. coli* (EPEC), and enteroaggregative *E. coli* (EAEC) and diffusely adherent *E. coli* (DAEC) [5,7]. The other important group is extraintestinal *E. coli* (ExPEC), which includes: avian pathogenic *E. coli* (APEC), neonatal meningitis *E. coli* (NMEC) and uropathogenic *E. coli* (UPEC) [5]. The strain *Escherichia coli* Nissle 1917 is a well-known probiotic and can be applied for the treatment of various dysfunctions and diseases of the intestinal tract [6]. It also has proven antimicrobial properties against certain pathogenic strains of *E. coli* This is especially important as several strains of *E. coli* apart from being pathogenic, are also known to have developed resistance to many antibiotics [8], and therapy with probiotics in certain cases of *E. coli* infections could be an alternative or adjuvant therapy to antibiotics.

The updated and grammatically correct definition of probiotics is “live microorganisms that, when administered in adequate amounts, confer a health benefit on the host” [9,10]. Probiotics are most commonly certain strains of lactic acid bacteria (LAB) that belong to the genera *Lactobacillus*, *Lactococcus*, *Pediococcus*, *Leuconostoc*, *Enterococcus*, *Streptococcus*, etc., as well as certain strains of bifidobacteria and the fungi *Saccharomyces boulardii* [10]. However other genera or species have also been defined as probiotics, such as *Escherichia coli* Nissle 1917 or certain strains of the genus *Bacillus* [11].

Until recently, the mainstream of research of probiotic effects was strain specific [10]. However, with accumulating evidence from cross-section studies on probiotic strains, their synergisms and interactions, some generalizations can be drawn that go beyond strain specific effects [10]. *Lactobacillus reuteri* DSM 17938 has proven effective antimicrobial activity, regulation of immune response as well as reduction of intestinal inflammation [12,13,14]; *Lactobacillus cassei* Shirota exerts favourable effects on colonic metabolism and alleviates symptoms of lactose intolerance [15,16]. Several strains of *Lactobacillus acidophilus* have proven to shorten hospitalisation of children with diarrhoea, reduce serum cholesterol, be effective as adjuvant therapy for bacterial vaginosis, and exhibit important immunomodulatory effects [17,18,19,20]. Different strains of *Bifidobacterium lactis* have effectively decreased symptoms of irritable bowel syndrome, constipation, dental plague, periodontopathogens and exhibited antimicrobial activity [21,22,23]. *Bacillus coagulans* GBI-30 is successful in reducing daily bowel movements in patients with irritable bowel syndrome [24]. The health benefits of various probiotics are clearly diverse, ranging from complicated host functions such as immune development, metabolic function or gut-brain interaction [25]. One of the important traits of probiotics is their antagonistic or antimicrobial effect against pathogens. This is achieved by competitive exclusion and/or the production of bacteriocins or bacteroicin-like substances and organic acids, thus regulating the intestinal microbiota [11,26]. Different single-strain or multi-strain probiotics have also proven to have an antagonistic effect against various pathogenic strains of *E. coli* [6,27,28,29,30].

Commercially available probiotics marketed as dietary supplements are often formulated as multi-strain products, whereas their survival and efficacy have often been investigated as single-strain products [31]. Therefore, it is important to determine if probiotic strains are antagonistic or synergistic towards each other or towards pathogens such as *E. coli*, when combined, which was the purpose of our research.

## 2. Materials and Methods

### 2.1. Probiotic Strains, Reference Microorganisms and Clinical Isolates

Five single-strain and four multi-strain probiotic dietary supplements supplied from pharmacies in Slovenia and Austria noted in Table 1 were used. Manufacturers’ names are omitted to enable impartiality of research.

To test the effect of probiotics to pathogenic strains of *E. coli*, different previously characterized clinical isolates of *E. coli*, obtained from the archive of the University of Ljubljana, Medical Faculty, Institute of Microbiology and Immunology, were used: EAEC (EAEC1, EAEC2 and EAEC3), EPEC (EPEC1 and EPEC2), ETEC (ETEC1) and ESBL-positive *E. coli* (ESBL1), The commensal strain *E. coli* K12 DSM 1562 obtained from the archive of the University of Maribor, Faculty of Health Sciences was used as a reference strain.

### 2.2. Enumeration of Microorganisms

Serial dilutions, ranging from 10 to 10^8^, were prepared. The colony counts of all fastidious probiotics was conducted according to ISO 4833:2013, part 2 using the surface plating technique and aerobic incubation on plate count agar at 30 °C. All strains and pathotypes of *E. coli* were incubated on MacConkey agar at 35 °C for 48 h.

### 2.3. Agar Spot Test

The antagonistic activities of the single-strain and multi-strain probiotics were determined using the modified agar spot method [27]. Briefly, 2 μL of each probiotic overnight culture (with final concentration 7 log cfu/mL) was inoculated onto De Man, Rogosa and Sharpe agar (MRS agar) except for SSP4 and SSP5, which were inoculated onto Mannitol Egg Yolk Polymyxin (MYP agar) and MacConkey agar, respectively. The plates were dried for 30 min at room temperature. All MRS agar plates were then incubated anaerobically at 35 °C for 24 h using anaerobic jars together with a Genbag anaerobic pouch. The MYP agar and MacConkey agar plates were incubated aerobically for 24 h. All plated were then overlaid with 10 mL of soft MacConkey agar inoculated with overnight cultures of the *E. coli* strains (with final concentration 7 log cfu/mL) and incubated at 35 °C for 48 h. After 48 h of incubation, measurements of inhibition zones around the LAB colonies were taken from the outer edge of the colonies to the outer edge of the clear zones. Inhibition zones of more than 20 mm, between 11 and 20 mm, and less than 10 mm were considered as strong, intermediate, and low inhibitions, respectively. Each test was performed in triplicate and the mean of the radii measuring from the edges of the colonies to the edges of the clear zones were calculated as well as the standard deviation SD.

### 2.4. Co-Culturing of Probiotic Strains and E. coli Strains in Milk

The modified co-culturing of the pathogens and the probiotics was conducted as follows: 20 mL of previously sterilised low-fat bovine milk was inoculated with one 500 μL of the overnight culture of the potential pathogen strain of *E. coli* (initial challenge between 10^7^ and 10^8^ cfu/mL) or with a mixture of the pathogen in the presence of 1 mL (initial challenge between 10^7^ and 10^8^ cfu/mL) of the overnight culture of the single-strain or multi-strain probiotic food supplements. The log step reduction was then calculated as RED = log [cfu_*E. coli*_/cfu_*E. coli*+probiotic_], where cfu_*E. coli*+probiotic_ is the count of cfu of *E. coli* after incubation of *E. coli* strains and probiotics in milk and cfu_*E. coli*_ is the count of cfu of *E. coli* after incubation of *E. coli* strains in milk. Fermentations were conducted with agitation (250 min^−1^) at 25 °C for 72 h. Two separate experiments were conducted and the mean was calculated for each sample [26,32].

### 2.5. DNA Detection of Probiotic Strains and E. coli Isolates

For the detection of bacterial strains, used in our assays, the specific PCR primers were used as shown in Table 2. Amplification was carried out in a thermal cycler (S Labcycler, Sensoquest, Goettingen, Germany), applying the cycling conditions as presented in Table 3. The reaction mixture (50 μL) contained 2.5 U of Taq DNA Polymerase (Qiagen, Hilden, Germany), 0.5 μM of each primer, 0.2 mM of each deoxyribonucleotide triphosphate, 1.5 mM of 10× reaction buffer, different concentrations of MgCl_2_ (2.5 mM MgCl_2_ (*Lactobacillus* genus, *Lactobacillus casei*, *Lactobacillus reuteri*), 2 mM MgCl_2_ (*Lactobacillus rhamnosus*, *Lactobacillus acidophilus*, *Bifidobacterium bifidum*), 1.5 mM MgCl_2_ (*Bifidobacterium* genus), 1 mM MgCl_2_ (*Bacillus coagulans*, *Enterococcus faecium*, *Escherichia coli*) and approx. 10 to 100 ng of bacterial DNA. All PCR products were stored at 4 °C until analysis.

Aliquots (5 μL) of the amplified products were subjected to electrophoresis (100 V, 45 min) in 1.5% agarose gels in TBE buffer (89 mM Tris base, 89 mM boric acid, 2 mM EDTA, pH 8.0). Gels were stained with 8 μL of Syber Green I and visualised under UV light at 312 nm. 

### 2.6. Statistical Analysis

The results were analysed using the IBM SPSS Statistics, version 24.0, software (IBM, New York, NY, USA). The independent *T*-test was used to compare the antagonistic effect of single-strain and multi-strain probiotic supplements on various strains on the inhibition of *E. coli* for both the agar spot test and the co-culturing method. The one-way ANOVA test was used to compare the influence of the inhibition of individual *E. coli* strains by probiotics as well as the influence of individual probiotics on the inhibition of the selected *E. coli* strains. The effects were considered statistically significant at 5% level of significance (*p* < 0.05).

## 3. Results

### 3.1. Agar-Spot Test

Mean zones of inhibition for five selected single-strain probiotics (SSP) and four multi-strain probiotics (MSP) using the agar-spot test are noted in Table 4. A statistically significant difference between the inhibition efficacy of the single-strain probiotics and the multi-strain probiotics against the selected *E. coli* strains was found using the independent group *T*-test (*p* < 0.05). The comparison of the inhibition of all probiotics against individual *E. coli* strains did not yield statistically significant differences using the one-way ANOVA test (*p* > 0.05). On the other hand, when comparing the efficacy of individual probiotics against the selected *E. coli* strains, a statistically significant difference was found using the one-way ANOVA test (*p* < 0.05). The most effective multi-strain probiotics were MSP1 and MSP4 with average inhibition zones of 7.04 mm and 6.12 mm respectively. The most effective probiotic was the multi-strain probiotic MSP1 which contains six lactic acid bacteria with an average inhibition zone of 7.04. As the inhibition zone did not reach more than 10 mm, the inhibition effect is classified as low [27]. Surprisingly the multi-strain probiotic MSP2 with a similar composition reached a mean of 4.77 mm. The most effective single-strain probiotic with an average inhibition zone of 6.64 mm was SSP3, which contains *Bifidobacterium animalis* subsp. *lactis* BB-12. The least effective single-strain probiotics were SSP4 and SSP5 with average inhibition zones of only 1.44 mm and 1.38 mm respectively. SSP4 and SSP5 contained *Bacillus coagulans* LMG S-24828 and *Escherichia coli* Nissle 1917, respectively.

### 3.2. Co-Culturing Method

Average log step reduction of cfu/mL *E. coli* strains incubated with four selected single-strain probiotics (SSP) and four multi-strain probiotics (MSP) in milk are noted in Table 5. The independent samples *T*-test revealed statistically significant differences between the cfu of *E. coli* strains co-cultured with single-strain probiotics and the multi-strain probiotics (*p* < 0.05) with this co-culturing method. However, the selected single-strain probiotics proved more effective as a higher mean log-step reduction at 1.35 of *E. coli* strains compared to the mean log-step reduction of *E. coli* strains co-cultured with the selected multi-strain probiotics at 0.69. 

A statistically significant difference using one-way ANOVA was not found for the log step reduction of the selected probiotics on different strains of *E. coli* (*p* > 0.05), thus again proving that there was no difference in the resistance of individual *E. coli* strains against probiotics. However, the co-culturing method revealed statistically significant differences between the antagonistic effect of the selected probiotics against all *E. coli* strains using one-way ANOVA (*p* < 0.05). 

The most effective probiotics proved to be SSP1, SSP3 and SSP2 with the mean log-step reduction of 1.76, 1.49 and 1.40 respectively. The probiotics contained *Lactobacillus reuteri* DSM 17938, *Bifidobacterium animalis* subsp. *lactis* BB-12 and *Lactobacillus casei* Shirota. The least effective single-strain probiotic was SSP4 which contained *Bacillus coagulans* LMG S-24828. The most effective multi-strain probiotic was MSP4 with a log-step reduction of 0.87. Statistically significant differences between the log step reduction of the growth of all strains of *E. coli* in milk compared with the growth of *E. coli* in milk in the presence of all selected probiotics was also found (*p* < 0.05) thus proving that all chosen probiotics had an antagonistic effect using the co-culturing method.

### 3.3. Identification of Species and Genera Using Molecular Methods

As noted in Table 6 using the PCR primer pairs LbLMA1-rev and R-16-1 [31], targeting the nucleotide sequence of the spacer between the 16S and 23S rRNA genes in a number of *Lactobacillus* strains, the genus *Lactobacillus* was confirmed by a positive band at 220 bp for all samples that contained lactobacilli (SSP1, SSP2, MSP1, MSP2, MSP3, MSP4). The genus *Bifidobacterium* was also confirmed for all bifidobacteria (SSP3, MSP2, MSP3, MSP4) using the primer pairs Bif164F and Bif601R for amplifying the 16S ribosomal rRNA fragments [34].

Several species-specific PCR were also conducted as obvious in Table 6. The multiplex PCR using species specific primer pairs noted in Table 2 [35] confirmed *Lactobacillus rhamnosus* in MSP1 and MSP2, *Lactobacillus acidophilus* in MSP1, MSP2 and MSP3 and *Bifidobacterium bifidum* in MSP2. However; *Lactobacillus acidophilus* was not confirmed in MSP4, which according to the manufacturer contains *Lactobacillus acidophilus* (species *L. gasseri*). On the other hand *Lactobacillus gasseri* was confirmed for this probiotic using the PCR primer pairs in Table 2 [37]. The duplex PCR using species specific primer pairs noted in Table 2 [36] confirmed *Lactobacillus casei* in SSP2 and MSP1 and *Lactobacillus reuteri* in SSP1 as well. A positive band 990 bp for *Bacillus coagulans* was found with the primer pairs BC1-F and BC2-R [38] in SSP3. *Enterococcus faecium* was also confirmed with the primer pairs EM1F and EM1R [39] in MSP1, MSP2 and MSP4. All *E. coli* strains were confirmed by PCR amplification with PCR primer pairs gadBF and gadBR [40]. The species-specific PCR protocols for the detection of the rest of the claimed probiotic species was not conducted.

## 4. Discussion

Molecular methods for the detection of probiotic microbes are very important since phenotype identification based on conventional culturing methods for the differentiation of probiotic microbes is time consuming and the selectivity of plate count media and fermentation test are insufficient to identify them, even at the genus level [33,36]. In our study two PCR protocols were used for genera specific PCR (*Lactobacillus* and *Bifidobacterium* spp.). The *Lactobacillus* species exhibited slight polymorphism as noted in the study by Dubernet and colleagues [33]. All probiotic supplements with these genera gave positive results. Although not every species-specific probiotic PCR was conducted, the main species were found by PCR as declared. This suggests that the reliability of the probiotic labelling system has improved [35]. The sample that, according to the manufacturer, claimed to contain *Lactobacillus acidophilus*, species *L. gasseri* did not give a positive band for *L. acidophilus*. However, this sample was positive for *L. gasseri;* which shows the importance of the usage of up-to-date and correct nomenclature. The *Lactobacillus acidophilus* complex include six separate species: *L. acidophilus*, *L. amylovorus*, *L. crispatus*, *L. gallinarum*, *L. gasseri and L. johnsonii* [41], thus the positive result for *L. gasseri* was correct. PCR thus proved to be an effective method for determining the probiotic strains and genera. Another important use of PCR is to detect the possible presence or transfer of antibiotic resistant genes [42] when adding probiotics and pathogens together, however this was not conducted in this study.

Antimicrobial activity against pathogens is an important attribute of probiotics for maintaining a healthy microbial balance in the gastrointestinal tract. The antagonistic activity of probiotic strains is mostly attributed to the production of metabolites such as organic acids, hydrogen peroxide, ethanol, acetaldehyde, acetoin, carbon dioxide, reuterin and other bacteriocins as well as competitive exclusion, immune modulation, stimulation of host defences and the production of signalling molecules that trigger changes in gene expression [26,27,43,44]. However correct methodology is important in order to determine realistic results. Most studies have been conducted using a single-strain probiotic and different chosen types of pathogenic bacteria using either the in vitro agar spot test or the well diffusion assay. Our study used two different methods for determining the antagonistic efficacy of probiotics—agar-spot test and co-culturing method—which presented different results.

Various modifications of the agar spot method have been used to prove that different *E. coli* strains have successfully inhibited various probiotics as seen from various studies. However, most studies included one chosen *E. coli* strain and single-strain probiotics. In the study Chapman and colleagues [45] various lactic acid bacteria, including *L. fermentum*, *L. plantarum*, *L. acidophilus*, *L. rhamnosus* and lactobacillus mixtures showed significant inhibition against *E. coli* NCTC 9001 with inhibition zones ranging from 5 mm to 20 mm. Brashears and colleagues [46] proved that 350 isolates of lactic acid bacteria showed significant inhibition against a four-strain mixture of *E. coli* O157:H7 with inhibition zones ranging from 1 mm to 11.5 mm. In the study by Shokryazdan and colleagues [27] the intermediate antagonistic effect of several lactic acid bacteria, including different strains of *Lactobacillus casei*, *Lactobacillus acidophilus*, *Lactobacillus fermentum* against different pathogens including *E. coli* ATCC 29181 an inhibition zone between 11.3 and 14.4 mm. Kholy and colleagues [29] also showed the efficiency of *Lactobacillus acidophilus*, *Lactobacillus plantarum* and *Bifidobacterium longum* to inhibit the growth of *E. coli* O157:H7 (inhibition diameter between 5 and 17 mm). In the study by Jacobsen and colleagues [28] of the tested 47 lactobacilli strains 30 exhibited an inhibition zone of up to 5 mm and above against *E. coli* (strain not specified). *Lactobacillus plantarum* P6 was also investigated for its antagonistic effect against *E. coli* ATCC 25921 in the study by Anas and colleagues [47]. It was also found that *Lactobacillus plantarum* P6 caused an inhibition zone of more than 2 mm against *E. coli* ATCC 25921. Another lactic acid bacteria belonging *Pediococcus acidilactici* FT28 also proved to have antagonistic activity against *E. coli* (strain not specified) as noted in the study Dowarah and colleagues [30]. *E. coli* MTCC 443 [48] and *E*. *coli* PTCC 1399 [49] were also inhibited by several lactobacillus isolates. *Lactobacillus rhamnosus* GG and bifidobacteria strains highly suppressed *E. coli* ATCC 700336 and ATCC 7000414 [50]. Our study using the agar spot test also proved that all chosen probiotics exhibited a moderate antagonistic effect against all *E. coli* strains as the mean inhibition zone for each probiotic ranged between 1.4 and 7.0 mm. The effect of individual probiotics was statistically significant (*p* < 0.05). The most effective probiotic was MSP1 (a mixture of two *Lactobacillus acidophilus* strains and one strain of each: *Lactobacillus casei*; *Lactobacillus plantarum*, *Lactobacillus rhamnosus*; *Enterococcus faecium*; *Lactococcus lactis*), followed by SSP3 (*Bifidobacterium animalis* subsp. *lactis*) and MSP4 (*Lactobacillus acidophilus* (species *L. gasseri*), *Bifidobacterium infantis*; *Enterococcus faecium*) with mean inhibition zones of 7 mm, 6.1 mm and 6.6 mm, respectively. 

On the other hand, our study showed no statistical significant differences (*p* > 0.05) of the inhibitory effect of any of the probiotics against an individual clinical pathogenic strain of *E. coli* (EAEC, EPEC, ETEC, ESBL) or the reference commensal strain *E. coli* K12. In fact, even the commensal reference strain did not prove to be less resistant to the antagonistic effect of the probiotics even though it lacked virulence factors. Also; the probiotic strain *E. coli* Nissle 1917 (SSP5) exhibited the lowest antagonistic effect against the pathogenic *E. coli* strains, thus showing that a commensal strain could compete with a pathogenic strain. However, it is unlikely that any particular commensal strain(s) of *E. coli* will be generally effective as a probiotic to prevent colonization by enteric pathogens, even though choosing *E. coli* as a probiotic would be consistent with its presumed ubiquitous presence in the gut [2,51]. The SSP4 (*Bacillus coagulans* LMG S-24828) was the second least effective probiotic against all strains of *E. coli*, thus coinciding with the research where different *Bacillus* species were less effective against the strain *E. coli* TISTR 887 [52]. Our study therefore supports all previously mentioned studies that different lactobacilli and bifidobacteria are most successful in inhibiting different *E. coli* strains or pathotypes. Although our study proved only moderate antagonistic effect (<10 mm) of all selected probiotics against all *E. coli* strains with the agar spot test in some articles it is not clear how the inhibition zone was measured, either as a diameter between two edges of the inhibition zone or as a radius measuring from the edge of the colony to the edge of the clear zone. The latter was used in our study. To avoid such differences as well as modifications of the agar spot test in most research conducted in this field, we recommend standardizing this method as the antagonistic effect of probiotics is an important trait. One possibility is to add to the antimicrobial susceptibility testing standard ISO 20776, which is generalized as the determination of susceptible, intermediate and resistant strains of bacteria to antimicrobial agents, a part specific for the antagonistic action of probiotics. 

Different methods of co-culturing have been used previously. The probiotic strain *Escherichia coli* Nissle 1917 has proven to be successful in decreasing the replication and survival of EHEC and EAEC strains using the co-culturing method with Caco-2 and LS-174T cell cultures [6]. Another co-culturing study [26] found that different fractions of *Lactobacillus acidophilus*- and *Lactobacillus casei*-fermented milk exhibited antimicrobial properties against *Escherichia coli* O157:H7, *Staphylococcus aureus*, *Enterococcus faecalis* and *Listeria innocua*. The co-culturing assay [30] investigated the antagonistic effect of lactobacilli isolates against several pathogens including *E. coli*. The pathogens were incubated in nutrient broth together with the chosen probiotics and compared to the count of *E. coli* in nutrient broth, which resulted in a 52% to 83% inhibition. Kholy and colleagues [29] studied the behaviour of *E. coli* O157:H7 during the fermentation and storage of probiotic yogurt (*L. acidophilus* La5 and *B. longum* ATCC 15707) and found that each probiotic decreased the viable population of *E. coli* for three log steps after 15 days. Similar results have also been found in the same type of co-culturing studies using different pathogens and probiotics [32,53]. Kholy and colleagues [29] also showed the log-step reduction of *E. coli* after incubation in milk with probiotics after three days’ fermentation in the range of 1–2 log steps, which is similar to our study. Our study thus proved all probiotics exhibited low antagonistic effect, with the single-strain probiotics being much more effective than the multi-strain probiotics. The most efficient probiotic species against all chosen *E. coli* strains by the co-culturing method were *Lactobacillus reuteri* DSM 17938, *Bifidobacterium animalis* subsp. *lactis* BB-12 and *Lactobacillus casei* Shirota with a log step reduction between 1.4 and 1.8. *Bacillus coagulans* and all multi-strain probiotics except MSP3 exhibited similar antagonistic effects (log step reduction between 0.7 and 0.9) against all strains of *E. coli*. The multi-strain probiotic MSP3 exhibited the lowest antagonistic effect (log step reduction 0.5). Perhaps the multi-strain probiotics exhibited interspecies competition for nutrients, as they were not put on specific nutrient agars such as agar MRS for lactobacilli, but were inoculated in milk with different composition of nutrients. Another possible explanation is that the co-culturing fermentation procedure in our study was conducted only for three days and that if it was conducted for a longer period, the results would be different. This also suggests that standardization of methods is very important in order to make comparisons.

Research into the greater functionality of multi-strain preparations compared to single-strain preparations has been inconclusive. The growth of urinary tract pathogens was inhibited by multi-strain probiotics, but they were not significantly more inhibitory than single-strains [45]. The study by Forssten and Ouwehand [31] also concluded that there were no substantial differences in reduction of levels of the same pathogenic strain when comparing single- and multi-strain products. However, there are studies that imply that multi-strain probiotics are more successful in inhibiting growth of pathogens than single-strains probiotics [32,54]. Our results show a statistically significant higher inhibitory efficacy of multi-strain probiotics against the chosen *E. coli* strains than the single-strain probiotics using the agar-spot test. However, the statistically significant differences found using the co-culturing method proved the exact opposite—that the single-strain probiotics were more successful against the chosen *E. coli* strains. In fact, all probiotics reached only a moderate inhibition effect as the average inhibition zone only reached up to 10 mm in the agar spot test and the average log step reduction of *E. coli* in the co-culturing method did not exceed 1.64 log steps. The most effective probiotic using the agar spot test was MSP1, whilst the most effective probiotic using the co-culturing method was SSP1 (*Lactobacillus reuteri* DSM 17938). On the other hand, the MSP3, with a similar composition as MSP1 did not prove a similar effectiveness. This could be due to different production or lyophilisation techniques by different producers that result in more or less effective probiotics or that the strain combination of one probiotic product was more efficient than the other. From our results it is also obvious that different methods do not give the same results and that an effective antagonistic effect with one in vitro method is not significant enough to make general assumptions of effect in real clinical conditions, where many different factors can contribute to the end result; therefore, critical assessment and of course double-blinded, placebo-controlled clinical trials are necessary before rendering an antagonistic effect of probiotics. In our case the co-culturing method with milk obviously influenced the multi-strain probiotics, and perhaps interspecies inhibition and competition occurred [54] or perhaps insufficient fermentation time occurred [29]. To determine the exact mechanisms further research is necessary.

## 5. Conclusions

The results of our study clearly indicate that although all chosen probiotics exhibited antagonistic effects against all selected clinical pathotypes of *E. coli* as well as against the commensal reference strain, the levels varied between the individual probiotics and were also dependent on the chosen in vitro method as the agar-spot test showed that the multi-strain probiotics were more effective than the single-strain probiotics and the co-culturing method showed the opposite result. The most effective single-strain probiotics against *E. coli* strains were *Bifidobacterium animalis* subsp. *lactis* BB-12 and *Lactobacillus reuteri* DSM 17938. The most effective multi-strain probiotics contained lactobacilli, bifidobacteria in enterococcus strains, thus proving that the lactic acid bacteria and bifidobacteria are most effective probiotics against *E. coli* strains. We can conclude that caution is necessary when choosing a probiotic supplement as not all have the same effectiveness. In vitro tests are therefore only the first step of predicting the effect of probiotics in the human gastrointestinal tract and further studies in the form of clinical trials are needed to determine the real efficacy of probiotics.

## Figures and Tables

**Table 1:** Characteristics of the probiotic supplements.

Probiotic Supplement *	Concentration of Microbes According to Manufacturer **	Microorganisms According to the Manufacturers	Intended Use
SSP1	10^8^ cfu/5 drops	*Lactobacillus reuteri* DSM 17938	Infant colic, restoring natural balance of microbiota in the intestine
SSP2	6.5 × 10^9^ cfu/65 mL	*Lactobacillus casei* Shirota	Improving gut health, gut function and immune modulation
SSP3	10^9^ cfu/5 drops	*Bifidobacterium animalis* subsp. *lactis* BB-12	Restoring balance of intestine microbiota, treating diarrhoea in infants
SSP4	2 × 10^9^ cfu/capsule	*Bacillus coagulans* LMG S-24828	For regulating intestinal microbiota
SSP5	2.5–25 × 10^9^ cfu/capsule	*Escherichia coli* Nissle 1917	For chronic constipation and ulcerative colitis in remission stage, treating diarrhoea, colonisation prophylaxis in infants
MSP1	3 × 10^9^ cfu/3 g	*Lactobacillus acidophilus* W22; *Lactobacillus casei* W56; *Lactobacillus plantarum* W62; *Lactobacillus rhamnosus* W71; *Lactobacillus salivarius* W57; *Enterococcus faecium* W54; *Lactococcus lactis* W58	Restoring balance of intestine microbiota, especially for overweight people
MSP2	5 × 10^9^ cfu/3 g	*Lactobacillus acidophilus* NIZO 3678; *Lactobacillus acidophilus* NIZO 3887; *Lactobacillus paracasei* NIZO 3672; *Lactobacillus plantarum* NIZO 3684; *Lactobacillus rhamnosus* NIZO 3689; *Lactobacillus salivarius* NIZO 3675; *Bifidobacterium bifidum* NIZO 3804; *Bifidobacterium lactis* NIZO 3680; *Enterococcus faecium* NIZO 3886	Restoring balance of intestine microbiota during consumption of antibiotics
MSP3	2 × 10^9^ cfu/7.5 mL	*Lactobacillus acidophilus* La-14; *Lactobacillus plantarum* Lp-115; *Lactobacillus paracasei* Lpc-37; *Bifidobacterium lactis* Bl-04	Restoring balance of intestine microbiota during consumption of antibiotics and constipation at home and during traveling for children
MSP4	1.2 × 10^7^ cfu/capsule	*Lactobacillus acidophilus* (species *L. gasseri*) PTA-5845; *Bifidobacterium infantis* PTA-5843; *Enterococcus faecium* PTA-5844	For treating bloating and diarrhoea and restoring balance of intestine microbiota

* SSP—Single-strain probiotic; MSP—multi-strain probiotic. ** The original concentration of probiotics according to the manufacturers.

**Table 2:** Primer pairs of selected probiotics.

Microorganism	Primer Pairs		Reference
*Lactobacillus* genus	LbLMA1-rev	CTC AAA ACT AAA CAA AGT TTC	[33]
	R-16-1	CTT GTA CAC ACC GCC CGT C	
*Bifidobacterium* genus	Bif164F	GGG TGG TAA TGC CGG ATG	[34]
	Bif601R	TAA GCC ATG GAC TTT CAC ACC	
*Lactobacillus rhamnosus*	Rham 1	GTC GAA CGA GTT CTG ATT ATT G	[35]
	RhamR	GAA CCA TGC GGT TCT TGG AT	
*Lactobacillus acidophilus*	LacidoF	CAC TTC GGT GAT GAC GTT GG	
	LacidoR	CGA TGC AGT TCC TCG GTT AAG C	
*Bifidobacterium bifidum*	BifF	ATT TGA GCC ACT GTC TGG TG	
	BifR	CAT CCG GGA ACG TCG GGA AA	
*Lactobacillus casei*	PrI	CAG ACT GAA AGT CTG ACG G	[36]
	CasII	GCG ATG CGA ATT TCT TTT TC	
*Lactobacillus reuteri*	Lfpr	GCC GCC TAA GGT GGG ACA GAT	
	Reu	AAC ACT CAA GGA TTG TCT GA	
*Lactobacillus gasseri*	Lgas-3	GCG ACC GAG AAG AGA GAG A	[37]
	Lgas-2	TGC TAT CGC TTC AAG TGC TT	
*Bacillus coagulans*	BC1-F	ACA GGG CTT TCA GAT ACC CG	[38]
	BC1-R	CGG GGA TCC GTC CAT CAA AA	
*Enterococcus faecium*	EM1F	TTG AGG CAG ACC AGA TTG ACG	[39]
	EM1R	TAT GAC AGC GAC TCC GAT TCC	
*Escherichia coli*	gadBF	ACC TGC GTT GCG TAA ATA	[40]
	gadBR	GGG CGG GAG AAG TTG ATG	

**Table 3:** Cycling parameters for polymerase chain reaction programs of selected probiotics.

Cycling Parameters *	PCR Program for *Lactobacillus* Genus	PCR Program for *Bifidobacterium* Genus	Multiplex Program for *L. rhamnosus*, *L. acidophilus*, *B. bifidum*	Duplex Program for *L. casei* and *L. reuteri*	PCR Program for *B. coagulans*	PCR Program for *E. faecium*	PCR Program for *E. coli*
Denaturation	30 s at 94 °C	30 s at 94 °C	45 s at 94 °C	30 s at 94 °C	30 s at 94 °C	60 s at 94 °C	30 s at 94 °C
Annealing	30 s at 55 °C	60 s at 53 °C	45 s at 63 °C	30 s at 55 °C	30 s at 60 °C	60 s at 54 °C	30 s at 52 °C
Extension	30 s at 72 °C	2 min at 72 °C	60 s at 72 °C	30 s at 72 °C	60 s at 72 °C	60 s at 72 °C	60 s at 72 °C
Number of cycles	20	35	30	30	30	40	35

* Initial denaturation and final extension is 15 min at 95 °C and 7 min at 72 °C respectively for all amplifications.

**Table 4:** Antagonistic effect of probiotics against selected strains of *E. coli* using the agar spot test.

*E. coli* Strain	Mean Inhibition Zone Diameter * (mm ± SD)								
	SSP1	SSP2	SSP3	SSP4	SSP5	MSP1	MSP2	MSP3	MSP4
EAEC1	2.50 ± 0.71	3.00 ± 0.71	9.67 ± 3.51	1.50 ± 0.58	1.00 ± 0.50	12.00 ± 2.00	4.00 ± 0.57	8.67 ± 1.50	5.00 ± 0.82
EAEC2	1.00 ± 0.50	1.50 ± 0.41	4.00 ± 0.53	1.50 ± 0.71	1.00 ± 0.63	8.00 ± 0.82	5.50 ± 1.93	9.00 ± 1.00	5.50 ± 0.71
EAEC3	2.00 ± 0.82	2.50 ± 0.41	5.00 ± 0.53	1.00 ± 0.66	1.00 ± 0.44	7.00 ± 1.50	6.50 ± 2.14	5.50 ± 0.71	7.50 ± 0.58
EPEC1	4.50 ± 0.71	3.50 ± 0.50	5.00 ± 0.50	1.00 ± 0.53	1.00 ± 0.41	10.33 ± 1.15	5.50 ± 1.87	3.50 ± 0.71	5.00 ± 0.50
EPEC2	3.50 ± 0.71	2.67 ± 0.47	6.5 ± 0.71	2.00 ± 0.30	1.00 ± 0.63	4.5 ± 0.71	3.17 ± 0.48	4.0 ± 1.41	5.50 ± 0.72
ETEC	2.00 ± 0.57	3.00 ± 0.82	9.00 ± 2.65	1.00 ± 0.20	2.00 ± 0.82	5.00 ± 1.41	3.50 ± 1.04	3.5 ± 0.71	5.50 ± 0.71
ESBL	5.00 ± 0.50	4.00 ± 1.00	5.0 ± 1.00	1.00 ± 0.35	3.00 ± 1.00	3.50 ± 0.71	5.50 ± 1.80	2.00 ± 0.50	5.50 ± 1.00
Ec K12	0.00 ± 0.00	1.67 ± 0.47	9.00 ± 1.41	2.50 ± 0.71	1.00 ± 0.63	6.00 ± 1.41	4.50 ± 1.18	2.00 ± 0.50	9.5 ± 0.71
Mean	2.05	2.73	6.64	1.44	1.38	7.04	4.77	4.77	6.12

* mean of three separate trials in mm, measured from the outer edge of the colony to the edge of the clear zone.

**Table 5:** Antagonistic effect of probiotics against selected strains of *E. coli* using the co-culturing method.

*E. coli* Strain	Average Log Step Reduction of cfu/mL of *E. coli* Strains after Three Day Co-Culturing in Milk with Probiotics *							
	SSP1	SSP2	SSP3	SSP4	MSP1	MSP2	MSP3	MSP4
EAEC1	1.87	1.08	0.63	1.27	0.23	0.22	0.34	1.02
EPEC1	2.23	1.40	2.20	1.39	0.79	0.09	0.33	0.05
ETEC	1.73	1.78	2.13	0.55	1.03	1.24	1.00	1.25
ESBL	1.00	1.64	1.53	0.39	0.24	0.62	0.40	1.35
Ec K12	1.97	1.12	0.95	0.40	1.37	1.30	0.30	0.68
mean	1.76	1.40	1.49	0.73	0.73	0.69	0.47	0.87

* average log step reduction.

**Table 6:** Detection of probiotics using genera-specific and species-specific PCR.

Probiotic Supplement	Microorganisms Claimed	PCR Confirmed	
		Genus	Species
SSP1	*Lactobacillus reuteri* DSM 17938	*Lactobacillus*	*L. reuteri*
SSP2	*Lactobacillus casei* Shirota	*Lactobacillus*	*L. casei*
SSP3	*Bifidobacterium animalis* subsp. *lactis* BB-12	*Bifidobacterium*	/
SSP4	*Bacillus coagulans* LMG S-24828	/	*B. coagulans*
SSP5	*Escherichia coli* Nissle 1917	/	*E. coli*
MSP1	*Lactobacillus acidophilus* W57; *Lactobacillus acidophilus* W22; *Lactobacillus casei* W56; *Lactobacillus rhamnosus* W71; *Enterococcus faecium* W54; and others	*Lactobacillus*	*L. acidophilus* *L. casei* *L. rhamnosus* *E. faecium*
MSP2	*Lactobacillus acidophilus* NIZO 3678; *Lactobacillus acidophilus* NIZO 3887; *Lactobacillus rhamnosus* NIZO 3689; *Bifidobacterium bifidum* NIZO 3804; *Enterococcus faecium* NIZO 3886; and others	*Lactobacillus Bifidobacterium*	*L. acidophilus* *L. rhamnosus* *B. bifidum* *E. faecium*
MSP3	*Lactobacillus acidophilus* La-14; *Bifidobacterium lactis* Bl-04; and others	*Lactobacillus Bifidobacterium*	*L. acidophilus*
MSP4	*Lactobacillus acidophilus* (species *L. gasseri*) PTA-5845; *Bifidobacterium infantis* PTA-5843; *Enterococcus faecium* PTA-5844	*Lactobacillus Bifidobacterium*	*L. gasseri* *E. faecium*
EAEC1	enteroaggregative *E. coli*	/	*E. coli*
EAEC2	enteroaggregative *E. coli*	/	*E. coli*
EAEC3	enteroaggregative *E. coli*	/	*E. coli*
EPEC1	enteropathogenic *E. coli*	/	*E. coli*
EPEC2	enteropathogenic *E. coli*	/	*E. coli*
ETEC	enterotoxigenic *E. coli*	/	*E. coli*
ESBL	*E. coli* that produces extended-spectrum *β*-lactamases	/	*E. coli*
Ec K12	*E. coli* K12 DSM 1562	/	*E. coli*
